# Virtual reality gait training versus non-virtual reality gait training for improving participation in subacute stroke survivors: study protocol of the ViRTAS randomized controlled trial

**DOI:** 10.1186/s13063-018-3165-7

**Published:** 2019-01-29

**Authors:** Ilona J. M. de Rooij, Ingrid G. L. van de Port, Johanna M. A. Visser-Meily, Jan-Willem G. Meijer

**Affiliations:** 1 Revant Rehabilitation Centres, Breda, The Netherlands; 2 Department of Rehabilitation, Physical Therapy Science and Sports, Brain Center Rudolf Magnus, University Medical Center Utrecht, Utrecht, The Netherlands; 3 Center of Excellence for Rehabilitation Medicine, Brain Center Rudolf Magnus, University Medical Center Utrecht, and De Hoogstraat Rehabilitation, Utrecht, The Netherlands

**Keywords:** Stroke, Rehabilitation, Gait, virtual reality

## Abstract

**Background:**

A stroke often results in gait impairments, activity limitations and restricted participation in daily life. Virtual reality (VR) has shown to be beneficial for improving gait ability after stroke. Previous studies regarding VR focused mainly on improvements in functional outcomes. As participation in daily life is an important goal for rehabilitation after stroke, it is of importance to investigate if VR gait training improves participation. The primary aim of this study is to examine the effect of VR gait training on participation in community-living people after stroke.

**Methods/design:**

The ViRTAS study comprises a single-blinded, randomized controlled trial with two parallel groups. Fifty people between 2 weeks and 6 months after stroke, who experience constraints with walking in daily life, are randomly assigned to the virtual reality gait training (VRT) group or the non-virtual reality gait training (non-VRT) group. Both training interventions consist of 12 30-min sessions in an outpatient rehabilitation clinic during 6 weeks. Assessments are performed at baseline, post intervention and 3 months post intervention. The primary outcome is participation measured with the Utrecht Scale for Evaluation of Rehabilitation-Participation (USER-P). Secondary outcomes are subjective physical functioning, functional mobility, walking ability, walking activity, fatigue, anxiety and depression, falls efficacy and quality of life.

**Discussion:**

The results of the study provide insight into the effect of VR gait training on participation after stroke.

**Trial registration:**

Netherlands National Trial Register, Identifier NTR6215. Registered on 3 February 2017.

**Electronic supplementary material:**

The online version of this article (10.1186/s13063-018-3165-7) contains supplementary material, which is available to authorized users.

## Background

Stroke is the third most common cause of disability worldwide [1]. Globally, 17 million people suffer from a stroke each year [2]. A stroke may lead to a wide range of impairments affecting sensory, motor, cognitive and visual function. Impairment in motor function of the legs, specifically, leads to commonly seen gait deficits following stroke [3, 4]. Approximately 50% of the people who regain ambulation after stroke experience difficulties with walking in the community, for example with terrain irregularity, changes in level, obstacle avoidance, walking far distances and performing secondary tasks, leading to limitations in walking in everyday life [5–7]. In addition, the ability to perform additional cognitive or motor tasks (i.e., dual tasks) during walking is often diminished after stroke [8, 9]. This ability is necessary to adapt to environmental changes while walking (e.g., stepping over an obstacle or crossing a street). Because of the experienced walking impairments, people after stroke are limited in performing daily life activities [10] and not able to participate optimally in the community [7]. Many people after stroke experience participation restrictions in daily life [7, 11, 12], which makes maximizing participation an important aspect of rehabilitation [13].

Recent research has increasingly focused on the use of virtual reality (VR) in stroke rehabilitation, including to enhance walking [14–16]. Rehabilitation interventions in virtual environments can manipulate practice conditions to engage motivation, motor control, cognitive processes and sensory feedback-based learning mechanisms [17]. Principles of motor learning can be well applied in VR training by providing goal-oriented, repetitive and varied practice that is adjusted to the abilities of the patient [18]. Also, real-time feedback provided by using motion capture-based VR can stimulate motor learning after brain injury [19, 20]. The adjustable practice conditions enable therapists to add dual tasks and unexpected situations so that patients can learn to adapt to environmental changes while walking. VR interventions to train gait frequently comprise treadmill training systems in combination with a screen or a head-mounted device to create an immersive environment [16].

Although multiple studies have promising results showing that gait training using VR can improve balance and walking ability after stroke [15, 16, 21, 22], longer-term follow-up and outcomes on the level of activity and participation are lacking [14, 15]. Currently, it is not known whether functional improvements in walking after a VR intervention are translated to real life by increasing activity and participation level. Because participation is one of the main priorities in rehabilitation care, it is of importance to investigate if VR gait training improves participation.

The primary aim of the ViRTAS (Virtual Reality Training After Stroke) study is to examine the effect of VR gait training on participation in community-living people between 2 weeks and 6 months after stroke. VR gait training is compared with a non-VR gait training consisting of conventional treadmill training and functional gait exercises. Both treadmill training and task-oriented gait exercises are commonly used rehabilitation interventions that have been demonstrated to be effective in people after stroke [23–25]. We hypothesize that VR gait training is a better training type for improving participation in subacute stroke survivors compared to non-VR gait training. In addition, we measure the effect on secondary outcome measures including subjective physical functioning, functional mobility, walking ability, walking activity, fatigue, anxiety and depression, falls efficacy and quality of life.

## Methods/design

### Study design

The study is a single-blinded, randomized controlled trial with two parallel groups that investigates the effects of VR gait training on participation, subjective physical functioning and walking activity in people after stroke. Participants are allocated to the virtual reality gait training (VRT) group or non-virtual reality gait training (non-VRT) group. The protocol is described according to the Standard Protocol Items: Recommendations for Interventional Trials (SPIRIT) Checklist for clinical trials [26] (see Additional file 1).

### Setting

The training sessions and assessments for the study are conducted in outpatient rehabilitation clinic, Revant Rehabilitation Centres, Breda, The Netherlands.

### Participants

Potential participants are included if they meet the following inclusion criteria: (1) diagnosed with stroke according to the World Health Organization (WHO) definition [27], (2) a time since stroke between 2 weeks and 6 months, (3) ability to walk without physical assistance for balance and coordination (i.e., patient may require verbal supervision or stand-by help from a person or may use a walking aid) (Functional Ambulation Category ≥ 3), (4) experiencing self-perceived constraints with walking in daily life, (5) living in the community and (6) age 18 to 80 years. Potential participants are excluded if they (1) have insufficient cognitive skills or understanding of the Dutch language to reliably answer simple questions (based on the impression of the researcher), (2) suffer from severe visual impairments, severe forms of ataxia or uncontrolled epileptic seizures or (3) currently suffer from orthopedic disorders or other co-morbidities that may limit walking ability. The last two criteria are verified with the participant and when needed checked in medical records.

### Recruitment and consent

Participants are primarily recruited from the rehabilitation center by their physician or physiotherapist who provide patients a brief description of the study. If patients are interested, the clinician obtains permission to pass contact details of the patient to the research team. The researcher then contacts the patient by telephone to give them more information about participation in the study and to verify whether all inclusion criteria are met (eligibility screening). After this contact with the researcher, potential participants can decide whether to participate. If patients are willing to participate, written informed consent is obtained and patient details are passed to an independent person for randomization. Besides recruitment from the rehabilitation center, participants are recruited via flyers at the neurology department of the local hospital, physiotherapy practices and general practices in the area. People after stroke are then invited to contact the research team by telephone call, email or post. All participants provide written informed consent, and anonymity is assured. The protocol of the ViRTAS study has been approved by the Medical Ethics Review Committee of Slotervaart Hospital and Reade, Amsterdam, The Netherlands (P1668, NL59737.048.16) and the study is registered in the Netherlands National Trial Register (NTR6215).

### Procedure

**Fig. 1:**
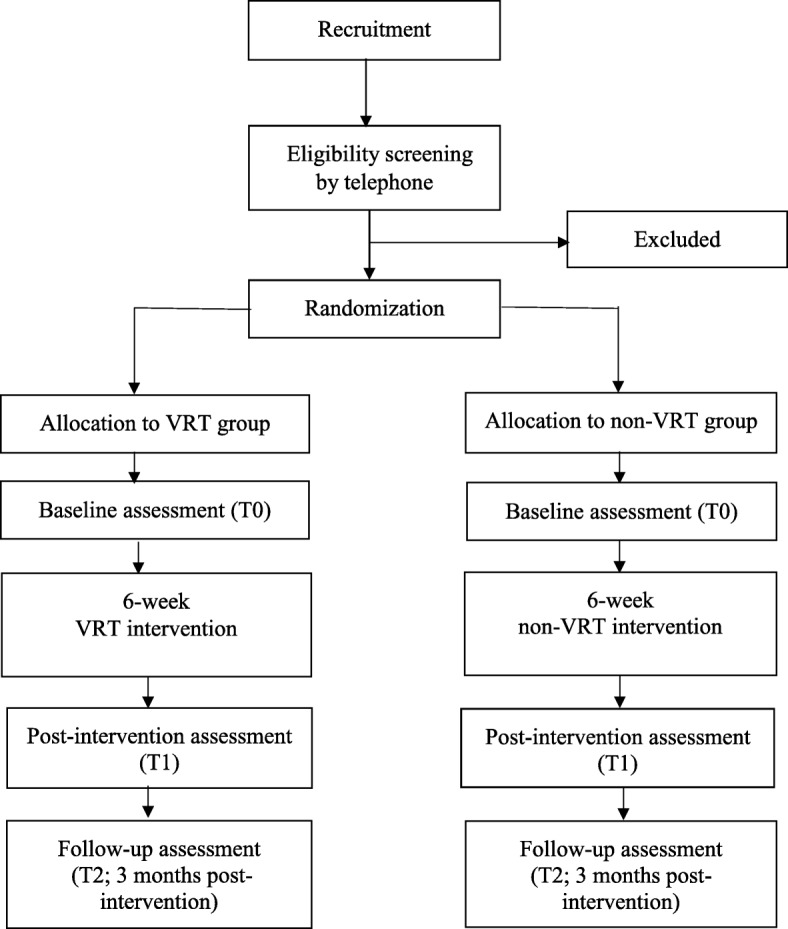
Flowchart of the study procedure

**Fig. 2:**
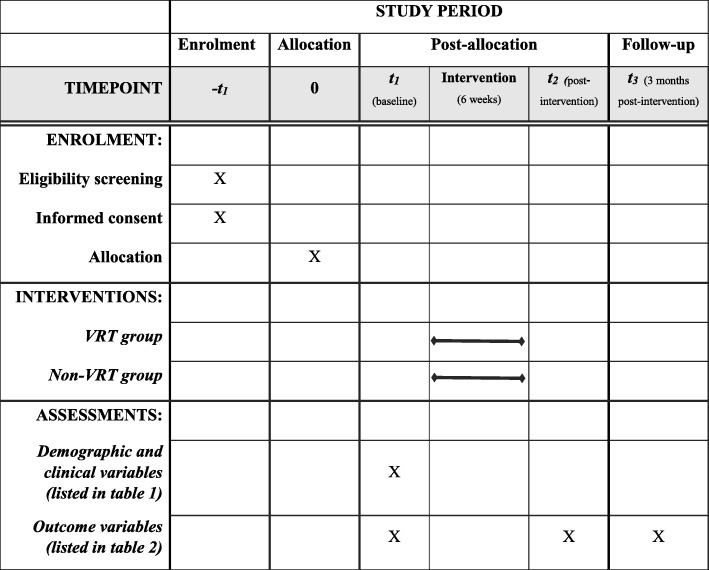
Standard Protocol Items: Recommendations for Interventional Trials (SPIRIT) schedule of enrollment, interventions and assessments

Participants in both the VRT and non-VRT group follow a training intervention of 2 30-min sessions per week for 6 weeks (12 sessions). Assessments are taken at baseline (T0), post intervention (T1, 6 weeks) and follow-up (T3, 3 months post intervention; Figs. 1 and 2). To promote participant retention, we plan training sessions in consultation with the participants and inform participants timely about the entire training schedule and the assessments. All outcomes are assessed in face-to-face meetings by the researcher (IdR). Data is collected on data collection forms, coded and entered into an electronic database by double data entry. The paper forms are stored in a locked cabinet and maintained for a period of 15 years. The researcher is responsible for the data management during the study. Adverse events (e.g., falls, pain and dizziness) that occur during the study period, whether or not related to the study intervention, are registered and in case of a serious adverse event the intervention will be discontinued for the participant. A serious adverse event is defined as an event that is fatal or life-threatening, requires hospital admission or extension of the admission, or causes invalidity or work disability. Participants in both groups continue to receive usual care and rehabilitation as provided by the rehabilitation center or other services in the area. The duration of gait-related therapies that participants visit parallel to the study intervention, are documented. Also, for each participant the adherence to the training sessions of the ViRTAS study is monitored by registering presence and any reasons for absence. In case the training intervention is discontinued for any reason, a participant is still requested to participate in the post intervention and follow-up assessments.

### Randomization and blinding

Participants are randomly assigned to the VRT group or the non-VRT group by an independent person who is not involved in the recruitment, intervention or assessments. The randomization is performed using sealed, opaque envelopes which contain a card stipulating to which group the participant is allocated. Twenty-five cards for both the VRT group and non-VRT group are placed in envelopes to ensure equal group sizes. The independent person picks a random envelope from the total set of envelopes and informs the participant and therapists about the treatment allocation. When randomization is done, the envelope is removed from the total set. The researcher who performs all assessments (IdR) is blinded to treatment allocation. Due to the nature of the intervention participants and physiotherapists providing the training intervention cannot be blinded to treatment allocation. Participants are explicitly asked not to disclose group allocation to the researcher. The assigned intervention is only revealed for the researcher when this is necessary to manage serious adverse events.

### Intervention group

**Fig. 3:**
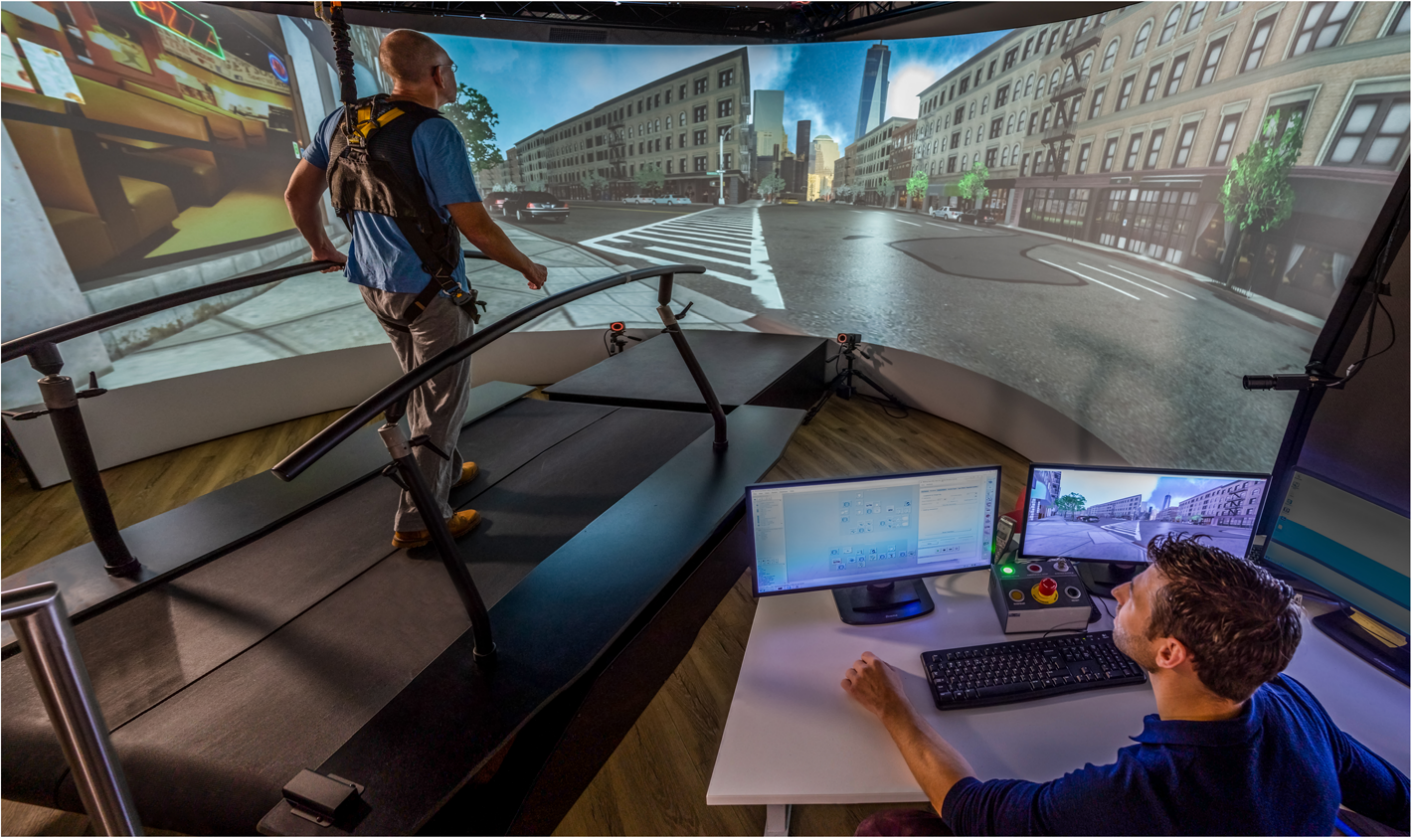
Setup of the virtual reality gait training intervention on the Gait Real-time Analysis Interactive Lab (GRAIL)

Participants who are allocated to the VRT group receive 2 30-min sessions of VRT on the Gait Real-time Analysis Interactive Lab (GRAIL, Motekforce Link, Amsterdam, The Netherlands) per week for 6 weeks (12 sessions). The GRAIL consists of a dual-belt treadmill with force platform, a motion-capture system (Vicon Motion Systems, Oxford, UK) and a 180° semi-cylindrical screen for the projection of environments with optic flow (Fig. 3) [28]. During the training sessions participants wear a safety harness that is attached to an overhead suspension system. This harness does not provide weight support. Specialized physiotherapists, who are certified for working with the GRAIL, choose, based on the therapeutic goals, which VR applications are used during the training sessions. Also, the physiotherapist regulates, based on the clinical expertise, the intensity of the exercises, decides the amount of progression and ensures that safety and quality of movement is maintained during the training. All applications can be individualized in terms of difficulty, for example by adjusting duration, speed, the amount of simultaneous tasks and the amount of real-time visual, auditory and/or tactile feedback during the exercises. The therapist records the settings of the VR applications and the perfomance of the participant.

### Comparison group

Participants assigned to the non-VRT group receive 2 30-min sessions of non-VRT per week for 6 weeks (12 sessions). The non-VRT consists of two stages: (1) conventional treadmill training (10–15 min) and (2) functional gait exercises (15 min). During the conventional treadmill training the speed is increased progressively. Also, the inclination angle of the treadmill may be increased. Functional gait exercises include six different exercises: (1) tapping or stepping up and down a step, (2) walking and picking up various objects from the ground, (3) walking on non-level surface, (4) walking a slalom, (5) stepping in hoops (increasing step length) and (6) stepping over a stick that is fixed between two pylons. The exercises are based on the exercises used in the FIT Stroke trial [29]. Training is guided by educated physiotherapists who can individualize the non-VRT. The therapists choose, based on the abilities and needs of the participants, which exercises are conducted during the different training sessions. Graded progression is achieved by increasing the difficulty of the tasks and increasing the number of repetitions. The exercises conducted in each training sessions are recorded by the physiotherapist.

### Outcome measures

During the baseline assessment several demographic, injury-related and therapy-related variables are identified. These variables are presented in Table 1.

**Table 1:** Baseline demographic and clinical variables

Demographic variables	
Age of patient at inclusion	
Gender	
Height	
Weight	
Partner	
Living situation	
Region of residence	
Injury-related clinical variables	
Time since stroke at inclusion	
Type of stroke	
Site of stroke	
Use of walking aids	
Use of orthoses	
Medication	
Co-morbidities	
Functional Ambulation Category score	
Therapy-related variables	
Duration of gait-related therapies parallel to study intervention	

An overview of the measurement instruments used to assess the primary and secondary outcome variables is given in Table 2.

**Table 2:** Outcome domains and measurement instruments

Outcome domain	Measurement instrument	Abbreviation	T0	T1	T2
Participation	Utrecht Scale for Evaluation of Rehabilitation-Participation	USER-P	X	X	X
Subjective physical functioning	Stroke Impact Scale-16	SIS-16	X	X	X
Fatigue	Fatigue Severity Scale	FSS	X	X	X
Anxiety and depression	Hospital Anxiety and Depression Scale	HADS	X	X	X
Falls efficacy	Falls Efficacy Scale International	FES-I	X	X	X
Quality of life	Stroke Specific Quality of Life Scale	SS-QOL	X	X	X
Walking ability	Six-minute walking test	6-MWT	X	X	X
Functional mobility	Timed-up & Go	TUG	X	X	X
Walking activity	Accelerometer monitoring (5 days)		X	X	X

T0: baseline, T1: post intervention (6 weeks), T2: follow-up (3 months post intervention)

#### Primary outcome

The effect of the intervention on participation is measured with the Utrecht Scale for Evaluation of Rehabilitation-Participation (USER-P). Participation can be defined as a person’s involvement in all life situations, whereby participation restrictions are problems one may experience in involvement in daily life situations [27, 30]. The USER-P assesses objective and subjective participation in persons with physical disabilities and covers three aspects of participation: Frequency, Restrictions and Satisfaction [31]. The Restrictions subscale of the USER-P is regarded as the primary outcome measure. The Restrictions subscale consists of 11 items and assesses the experienced participation restrictions in daily life activities including vocational, leisure and social activities. For example, “Does your stroke currently limit you in performing outdoor mobility?” Scores consists of NA (not applicable), not possible (1), with assistance (2), with difficulty (3) and without difficulty (4). The total score is calculated by the sum of all items converted into a 0–100 scale. A higher total score indicates less experienced restrictions [31]. The USER-P has satisfactory reproducibility [32], high responsiveness [33] and good construct, concurrent and discriminative validity [31].

#### Secondary outcomes

##### Frequency and Satisfaction scales of the USER-P

The Frequency subscale of the USER-P is divided in parts A and B. Part A measures the time that an individual has spent on paid work, unpaid work, volunteer work and housekeeping using scores from 0 (not at all) up to 5 (36 h or more). Part B registers the frequency of leisure and social activities in the past 4 weeks with scores ranging from 0 (not at all) to 5 (19 times or more). Furthermore, the Satisfaction subscale measures how satisfied someone is with vocational activities, leisure activities and social relationships. Items are scored on a scale of 0 (very dissatisfied) to 4 (very satisfied). The sum scores for the Frequency and Satisfaction scales are converted into a 0–100 scale. Higher scores represent a higher frequency and satisfaction [31].

##### Stroke Impact Scale-16 (SIS-16)

The SIS-16 is a stroke-specific instrument for measuring subjective physical functioning and consists of 16 from the 28 items of the physical domain of the original SIS version 3.0. The items are scored on a 5-point scale, from “not difficult at all” to “cannot do it at all.” The SIS-16 is an appropriate instrument to monitor physical limitations over time in subacute patients after stroke. The SIS-16 demonstrates good instrument reliability and concurrent validity [34].

##### Timed-up & Go (TUG)

The TUG measures functional mobility [35]. Participants are asked to rise from an armchair, walk 3 m, turn around, walk back and return to sitting [36]. The TUG has a high degree of reliability and validity when applied in people after stroke [37, 38]. Participants are allowed to use walking aids and/or ankle-foot orthosis if necessary. The TUG is performed three times to determine a mean test time.

##### Six-minute walking test (6-MWT)

The 6-MWT is a commonly used valid and reliable test to assess walking ability in people after stroke [39]. Participants are instructed to walk as far as possible at comfortable, but fast pace for 6 min. Distance walked in 6 min is assessed in a 40 m-long testing corridor with marking per 5 m. Each minute, participants are told how much time has elapsed or is left to complete the test. During the test participants are allowed to stand still or sit on a chair if they feel a need to rest.

##### Fatigue Severity Scale (FSS)

The FSS measures the level of fatigue and the impact of fatigue on daily functioning. This questionnaire consists of 9 items that are scored on a 7-point scale from 1 (completely disagree) to 7 (completely agree). The total score is calculated by the mean of the nine items [40]. Fatigue prevalence can be defined using a FSS score ≥ 4 [41]. The FSS has satisfactory internal reliability and validity [42].

##### Hospital Anxiety and Depression Scale (HADS)

The HADS is used to assess anxiety and depression. This questionnaire consists of 14 items (seven anxiety, seven depression) and all items are scored on a 4-point scale from 0 to 3 [43]. In the literature, a cutoff score of > 7 for both subscales is defined for the identification of depressive symptoms and symptoms of anxiety [44]. The HADS is a reliable and valid instrument that is sensitive over time [45].

##### Falls Efficacy Scale International (FES-I)

The FES-I consists of 16 items about the person’s level of confidence in avoiding falling during essential, non-hazardous activities of daily living [46]. The score of this instrument can range from 16 to 64, with higher scores indicating greater fear of falling or lower fall-related self-efficacy. The FES-I has good psychometric properties in older people [47] and people after stroke [48].

##### Stroke Specific Quality of Life Scale (SS-QOL)

The SS-QOL is used to measure quality of life. This questionnaire is designed for use in clinical stroke trials and consists of 49 items divided over 12 domains: energy, family roles, language, mobility, mood, personality, self-care, social roles, thinking, upper extremity function, vision and work/productivity. Each item is scored on a 5-point Likert scale and a total score is calculated by a mean of the 12 domains [49]. The SS-QOL has good test-retest reliability, internal consistency and validity in people after stroke [50, 51].

##### Activity monitoring

Participants wear a tri-axial accelerometer (DynaPort MM, McRoberts BV, The Hague, The Netherlands) to measure daily-life walking activity. The accelerometer (55 g) is worn for five consecutive days at baseline (T0), post intervention (T1) and follow-up (T2; 3 months post intervention). Five consecutive days of monitoring are necessary to obtain reliable walking activity data [52]. The measurement period includes always 1 or 2 weekend day(s). The device is placed at the middle of the lower back using an electric strap and can be worn above or underneath the clothes. Participants are preferably monitored during day and night but are allowed to take off the accelerometer during night time. During water-related activities such as swimming and showering, the accelerometer is removed to prevent water damage.

##### Intensity of training sessions

To monitor the intensity of the training sessions in both the VRT and non-VRT group the BORG-RPE scale (CR-10) and a pedometer (Digi-Walker SW-200, Yamax Corporation, Tokyo, Japan) were used. The BORG-RPE scale (CR-10) asks participants about the rate of perceived exertion and workload during the training sessions and can be scored from 0 (no exertion) to 10 (maximal exertion) [53, 54]. This score is noted at the start and the end of a training session. In addition, participants wear a pedometer on the waistband on the non-hemiplegic side during the training to measure the number of steps taken in the training sessions.

### Sample size

The sample size calculations are based on the primary outcome measure, the USER-P Restrictions subscale. A difference of 18.2 points on the USER-P Restrictions subscale is regarded as clinically relevant [32]. The standard deviation of the population is estimated at 17.9 points and the test-retest reliability (ICC) is suggested to be 0.85 [32]. Based on an alpha of 0.05 and a power of 80%, a minimum of 14 participants per group is necessary [55]. However, a relative high clinical relevant difference of 18.2 points (18%) is not expected in this study. Therefore, we re-estimated the sample size based on a difference of 15% (15 points) on the USER-P Restrictions subscale, resulting in a minimum of 21 participants per group [55]. Expecting a dropout rate of 20%, we assume that a minimum of 50 participants is needed to achieve a sufficient statistical power of 80%. The majority of the randomized studies regarding the effect of VR that are published up to now included less than 25 participants per group.

### Data analysis

Gait activity data monitored with the accelerometer is analyzed using a validated stroke-specific algorithm for gait detection and gait quantification in Matlab (The MathWorks Inc., Natick, MA, USA) [56]. This algorithm has shown to have good criterion validity and test-retest reliability in people after stroke. The algorithm detects gait activity with a minimum length of 8 s or a multiple of 8 s.

The effectiveness of the intervention on the primary outcome measure, USER-P Restrictions subscale, is assessed using random coefficient analysis. We include time of assessment, group assignment (intervention and comparison group) and the interaction between time of assessment and group assignment in the multi-level regression model. Because random coefficient analysis can handle missing data, the analysis is performed with all available data, including data from participants with incomplete datasets [57]. Intention-to-treat analysis will be applied. Also, for the secondary outcome measures (USER-P Frequency subscale, USER-P Satisfaction subscale, SIS-16, TUG, 6-MWT, FSS, HADS, FES-I, SS-QOL and gait activity) a comparable random coefficient analysis is performed to assess the effectiveness of the intervention. Demographic, injury-related and therapy-related variables of the 2 groups are examined using the independent *t* test or non-parametric equivalent, the Mann-Whitney *U* test. A *Χ*^2^ test is used to examine categorical variables. Furthermore, to compare the intensity of the training sessions in the VRT and non-VRT groups, the mean number of steps measured with the pedometer and the mean BORG-RPE score are analyzed with an independent *t* test or the non-parametric equivalent. Results are considered significant when *P* values are < 0.05.

## Discussion

The ViRTAS study examines the effect of VR gait training on participation in community-living people between 2 weeks and 6 months after stroke. Also, the effect of VR gait training on secondary outcome measures including subjective physical functioning, functional mobility, walking ability, walking activity, fatigue, anxiety and depression, falls efficacy and quality of life is discussed.

VR can be defined as a computer-based technology that simulates a real environment and provides the user with opportunities to interact with objects and events [20, 58]. In this study the VR consists of high-end three-dimensional environments with motion capture. VR is thought to enhance neuroplasticity and motor learning after stroke through facilitating brain reorganization and activating brain areas involved in motor planning, learning and execution [59]. Multiple studies have shown significant improvements in functional outcome measures as a result of VR gait training in people after stroke [15, 16]. We believe that VR gait training for subacute stroke survivors is a valuable addition to conventional physiotherapy (e.g., treadmill training or functional gait exercises) by providing an intensive, variable and enjoyable therapy which can be easily adjusted to the abilities of the patient. Multiple principles of motor learning can effectively be applied during a VR gait training session [18, 60]. Using VR gives the opportunity to perform multiple repetitions of different movements within meaningful tasks by varying the gait exercises and the settings of the exercises within a training session. Variability in training is thought to be important for retention and transfer of learned skills [61]. While walking in a virtual environment unexpected constraints (e.g., disturbances and obstacle avoidance) or dual tasks can be provided to stimulate patients to use problem-solving abilities. This is useful as it is known that problem-solving is an important principle to enhance the cognitive learning of new skills [17]. Using VR, environments can be manipulated more than during conventional physiotherapy. In addition, the use of enriched VR environments with game scores and a high virtual presence can improve motivation, enjoyment and engagement of patients probably more than during conventional physiotherapy interventions [62, 63]. Another advantage of VR compared to conventional therapy could be that the task difficulty can easily be monitored with multiple options present to adjust the training to the abilities of the individual. Lastly, intrinsic and extrinsic feedback (knowledge of performance and knowledge of results) provided during a VR gait training session can promote motor learning after stroke [19]. In this study, we investigate whether these above-mentioned potentially beneficial characteristics of VR can lead to improvement in participation in people after stroke. We match both training interventions on frequency, duration and number of training sessions.

A foreseen difficulty of the study is that participants continue to receive usual care and rehabilitation which might interfere with the effect of the studied interventions. From an ethical perspective, it is not an option to withheld care or rehabilitation from subacute stroke survivors. As participants are within 6 months after stroke, they may receive other therapies focusing on gait. However, due to the randomization it is expected that there is no noticeable difference between the VRT and non-VRT group in the potential interference of usual care and rehabilitation. Still, the frequency and duration of the gait-related therapies are registered.

To the authors’ knowledge this is the first study to investigate the effect of a VR gait training intervention on the level of participation in people after stroke. VR gait training might be a great potential for rehabilitation after stroke.

## Trial status

The trial is still ongoing. The first participant was included in April 2017 and the patient recruitment will be completed around August 2019.

## Additional file

Additional file 1: Standard Protocol Items: Recommendations for Interventional Trials (SPIRIT) 2013 Checklist: recommended items to address in a clinical trial protocol and related documents. (DOC 125 kb)

## Abbreviations

6-MWT: Six-minute walking test
FES-I: Falls Efficacy Scale International
FSS: Fatigue Severity Scale
GRAIL: Gait Real-time Analysis Interactive Lab
HADS: Hospital Anxiety and Depression Scale
SIS-16: Stroke Impact Scale-16
SS-QOL: Stroke Specific Quality of Life Scale
TUG: Timed-up & Go
USER-P: Utrecht Scale for Evaluation of Rehabilitation-Participation
ViRTAS: Virtual Reality Training After Stroke
VR: Virtual reality
VRT: Virtual reality gait training

## References

[1] Feigin VL, Norrving B, Mensah GA (2017). Global Burden of Stroke. Circ Res.

[2] World Stroke Organization. Annual Report 2016. 2016. https://www.world-stroke.org/images/Annual_Report_2016_online.pdf. Accessed 29 Aug 2017.

[3] Langhorne P, Coupar F, Pollock A (2009). Motor recovery after stroke: a systematic review. Lancet Neurol.

[4] Pollock C, Eng J, Garland S (2011). Clinical measurement of walking balance in people post stroke: a systematic review. Clin Rehabil.

[5] Jørgensen HS, Nakayama H, Raaschou HO, Olsen TS (1995). Recovery of walking function in stroke patients: the Copenhagen Stroke Study. Arch Phys Med Rehabil.

[6] Perry J, Garrett M, Gronley JK, Mulroy SJ (1995). Classification of walking handicap in the stroke population. Stroke.

[7] Mayo NE, Wood-Dauphinee S, Côté R, Durcan L, Carlton J (2002). Activity, participation, and quality of life 6 months poststroke. Arch Phys Med Rehabil.

[8] Plummer-D'Amato P, Altmann LJ, Saracino D, Fox E, Behrman AL, Marsiske M (2008). Interactions between cognitive tasks and gait after stroke: a dual task study. Gait Posture.

[9] Yang L, Lam FM, Liao LR, Huang MZ, He CQ, Pang MY (2017). Psychometric properties of dual-task balance and walking assessments for individuals with neurological conditions: a systematic review. Gait Posture.

[10] Field MJ, Gebruers N, Shanmuga Sundaram T, Nicholson S, Mead G (2013). Physical activity after stroke: a systematic review and meta-analysis. ISRN Stroke.

[11] van der Zee CH, Visser-Meily JM, Lindeman E, Jaap Kappelle L, Post MW (2013). Participation in the chronic phase of stroke. Top Stroke Rehabil.

[12] de Graaf JA, van Mierlo ML, Post MWM, Achterberg WP, Kappelle LJ, Visser-Meily JMA. Long-term restrictions in participation in stroke survivors under and over 70 years of age. Disabil Rehabil. 2018;40(6):637-45. 10.1080/09638288.2016.1271466 28054834

[13] Zahuranec DB, Skolarus LE, Feng C, Freedman VA, Burke JF (2017). Activity limitations and subjective well-being after stroke. Neurology.

[14] Laver KE, Lange B, George S, Deutsch JE, Saposnik G, Crotty M. Virtual reality for stroke rehabilitation. Cochrane Database Syst Rev. 2017;(11):CD008349. 10.1002/14651858.CD008349.pub4 PMC6485957 29156493

[15] de Rooij IJM, van de Port IGL, Meijer J-WG (2016). Effect of virtual reality training on balance and gait ability in patients with stroke: systematic review and meta-analysis. Phys Ther.

[16] Corbetta D, Imeri F, Gatti R (2015). Rehabilitation that incorporates virtual reality is more effective than standard rehabilitation for improving walking speed, balance and mobility after stroke: a systematic review. J Physiother.

[17] Levin MF, Weiss PL, Keshner EA (2015). Emergence of virtual reality as a tool for upper limb rehabilitation: incorporation of motor control and motor learning principles. Phys Ther.

[18] Langhorne P, Bernhardt J, Kwakkel G (2011). Stroke rehabilitation. Lancet.

[19] Winstein CJ, Merians AS, Sullivan KJ (1999). Motor learning after unilateral brain damage. Neuropsychologia.

[20] Holden MK (2005). Virtual environments for motor rehabilitation: review. CyberPsychol Behav.

[21] Luque-Moreno C, Ferragut-Garcías A, Rodríguez-Blanco C, Heredia-Rizo AM, Oliva-Pascual-Vaca J, Kiper P, Oliva-Pascual-Vaca Á (2015). A decade of progress using virtual reality for poststroke lower extremity rehabilitation: systematic review of the intervention methods. Biomed Res Int.

[22] Darekar A, McFadyen BJ, Lamontagne A, Fung J (2015). Efficacy of virtual reality-based intervention on balance and mobility disorders post-stroke: a scoping review. J Neuroeng Rehabil.

[23] Mehrholz J, Pohl M, Elsner B. Treadmill training and body weight support for walking after stroke. Cochrane Database Syst Rev. 2017;(8):CD002840. 10.1002/14651858.CD002840.pub4 PMC6483714 28815562

[24] Eng JJ, Tang PF (2007). Gait training strategies to optimize walking ability in people with stroke: a synthesis of the evidence. Expert Rev Neurother.

[25] Wevers L, van de Port I, Vermue M, Mead G, Kwakkel G (2009). Effects of task-oriented circuit class training on walking competency after stroke: a systematic review. Stroke.

[26] Chan AW, Tetzlaff JM, Gøtzsche PC, Altman DG, Mann H, Berlin JA, Dickersin K, Hrobjartsson A, Schulz KF, Parulekar WR, Krleza-Jeric K, Laupacis A, Moher D (2013). SPIRIT 2013 explanation and elaboration: guidance for protocols of clinical trials. BMJ.

[27] WHO (2001). International Classification of Functioning, Disability and Health: ICF.

[28] de Rooij IJM, van de Port IGL, Meijer J-WG (2017). Feasibility and effectiveness of virtual reality training on balance and gait recovery early after stroke: a pilot study. Int J Phys Med Rehabil.

[29] van de Port IG, Wevers L, Roelse H, van Kats L, Lindeman E, Kwakkel G (2009). Cost-effectiveness of a structured progressive task-oriented circuit class training programme to enhance walking competency after stroke: the protocol of the FIT-Stroke trial. BMC Neurol.

[30] Dahl TH (2002). International classification of functioning, disability and health: an introduction and discussion of its potential impact on rehabilitation services and research. J Rehabil Med.

[31] Post MW, van der Zee CH, Hennink J, Schafrat CG, Visser-Meily JM, van Berlekom SB (2012). Validity of the Utrecht Scale for Evaluation of Rehabilitation-Participation. Disabil Rehabil.

[32] van der Zee CH, Priesterbach AR, van der Dussen L, Kap A, Schepers VP, Visser-Meily JM, Post MW (2010). Reproducibility of three self-report participation measures: The ICF Measure of Participation and Activities Screener, the Participation Scale, and the Utrecht Scale for Evaluation of Rehabilitation-Participation. J Rehabil Med.

[33] van der Zee CH, Kap A, Rambaran Mishre R, Schouten EJ, Post MW (2011). Responsiveness of four participation measures to changes during and after outpatient rehabilitation. J Rehabil Med.

[34] Duncan PW, Lai SM, Bode RK, Perera S, DeRosa J (2003). Stroke Impact Scale-16: a brief assessment of physical function. Neurology.

[35] Shumway-Cook A, Brauer S, Woollacott M (2000). Predicting the probability for falls in community-dwelling older adults using the Timed Up & Go Test. Phys Ther.

[36] Podsiadlo D, Richardson S (1991). The Timed “Up & Go”: a test of basic functional mobility for frail elderly persons. J Am Geriatr Soc.

[37] Ng SS, Hui-Chan CW (2005). The Timed Up & Go test: its reliability and association with lower-limb impairments and locomotor capacities in people with chronic stroke. Arch Phys Med Rehabil.

[38] Chan PP, Si Tou JI, Tse MM, Ng SS (2017). Reliability and validity of the Timed Up and Go Test with a motor task in people with chronic stroke. Arch Phys Med Rehabil.

[39] Fulk GD, Echternach JL, Nof L, O’Sullivan S (2008). Clinometric properties of the six-minute walk test in individuals undergoing rehabilitation poststroke. Physiother Theory Pract.

[40] Krupp LB, LaRocca NG, Muir-Nash J, Steinberg AD (1989). The Fatigue Severity Scale. Application to patients with multiple sclerosis and systemic lupus erythematosus. Arch Neurol.

[41] Cumming TB, Packer M, Kramer SF, English C (2016). The prevalence of fatigue after stroke: a systematic review and meta-analysis. Int J Stroke.

[42] Lerdal A, Kottorp A (2011). Psychometric properties of the Fatigue Severity Scale-Rasch analyses of individual responses in a Norwegian stroke cohort. Int J Nurs Stud.

[43] Zigmond AS, Snaith RP (1983). The Hospital Anxiety and Depression Scale. Acta Psychiatr Scand.

[44] Aben I, Verhey F, Lousberg R, Lodder J, Honig A (2002). Validity of the Beck Depression Inventory, Hospital Anxiety and Depression Scale, SCL-90, and Hamilton Depression Rating Scale as screening instruments for depression in stroke patients. Psychosomatics.

[45] Herrmann C (1997). International experiences with the Hospital Anxiety and Depression Scale-A review of validation data and clinical results. J Psychosom Res.

[46] Yardley L, Beyer N, Hauer K, Kempen G, Piot-Ziegler C, Todd C (2005). Development and initial validation of the Falls Efficacy Scale-International (FES-I). Age Ageing.

[47] Kempen GI, Todd CJ, Van Haastregt JC, Zijlstra GA, Beyer N, Freiberger E, Hauer KA, Piot-Ziegler C, Yardley L (2007). Cross-cultural validation of the Falls Efficacy Scale International (FES-I) in older people: results from Germany, the Netherlands and the UK were satisfactory. Disabil Rehabil.

[48] Azad A, Hassani Mehraban A, Mehrpour M, Mohammadi B (2014). Clinical assessment of fear of falling after stroke: validity, reliability and responsiveness of the Persian version of the Fall Efficacy Scale-International. Med J Islam Repub Iran.

[49] Williams LS, Weinberger M, Harris LE, Clark DO, Biller J (1999). Development of a Stroke-Specific Quality of Life Scale. Stroke.

[50] Muus I, Williams LS, Ringsberg KC (2007). Validation of the Stroke Specific Quality of Life Scale (SS-QOL): test of reliability and validity of the Danish version (SS-QOL-DK). Clin Rehabil.

[51] Boosman H, Passier PE, Visser-Meily JM, Rinkel GJ, Post MW (2010). Validation of the Stroke Specific Quality of Life scale in patients with aneurysmal subarachnoid haemorrhage. J Neurol Neurosurg Psychiatry.

[52] van Schooten KS, Rispens SM, Elders PJ, Lips P, van Dieen JH, Pijnappels M (2015). Assessing physical activity in older adults: required days of trunk accelerometer measurements for reliable estimation. J Aging Phys Act.

[53] Borg GA (1982). Psychophysical bases of perceived exertion. Med Sci Sports Exerc.

[54] Gauthier LV, Kane C, Borstad A, Strahl N, Uswatte G, Taub E, Morris D, Hall A, Arakelian M, Mark V (2017). Video Game Rehabilitation for Outpatient Stroke (VIGoROUS): protocol for a multi-center comparative effectiveness trial of in-home gamified constraint-induced movement therapy for rehabilitation of chronic upper extremity hemiparesis. BMC Neurol.

[55] Twisk JWR, Twisk JWR (2003). Sample size calculations. Applied longitudinal data analysis for epidemiology: a practical guide.

[56] Punt M, van Alphen B, van de Port IG, van Dieën JH, Michael K, Outermans J, Wittink H (2014). Clinimetric properties of a novel feedback device for assessing gait parameters in stroke survivors. J Neuroeng Rehabil.

[57] Twisk JWR (2004). Longitudinal data analysis. A comparison between generalized estimating equations and random coefficient analysis. Eur J Epidemiol.

[58] Wilson PN, Foreman N, Stanton D (1997). Virtual reality, disability and rehabilitation. Disabil Rehabil.

[59] Calabrò RS, Naro A, Russo M, Leo A, De Luca R, Balletta T, Buda A, La Rosa G, Bramanti A, Bramanti P (2017). The role of virtual reality in improving motor performance as revealed by EEG: a randomized clinical trial. J Neuroeng Rehabil.

[60] French B, Thomas LH, Leathley MJ, Sutton CJ, McAdam J, Forster A, Langhorne P, Price CI, Walker A, Watkins CL. Repetitive task training for improving functional ability after stroke. Cochrane Database Syst Rev. 2007;(4):CD006073. 10.1002/14651858.CD006073.pub2 17943883

[61] Beek PJ, Roerdink M, Selzer ME, Clarke S, Cohen LG, Kwakkel G, Miller RH (2014). Evolving insights into motor learning and their implications for neurorehabilitation. Textbook of neural repair and rehabilitation.

[62] Sveistrup H (2004). Motor rehabilitation using virtual reality. J Neuroeng Rehabil.

[63] Proffitt R, Lange B (2015). Considerations in the efficacy and effectiveness of virtual reality interventions for stroke rehabilitation: moving the field forward. Phys Ther.

